# Samaritan Free Hospital New Building

**Published:** 1905-06-24

**Authors:** 


### SAMARITAN FREE HOSPITAL NEW BUILDING.

A fashionable and crowded gathering met at the opening ceremonial on June 14th of the new out-patient department of the Samaritan Hospital. In the unavoidable absence at Windsor of Viscount Portman, his place was taken by Lord Leigh, who formally declared the building open, and in expressing the great pleasure it gave him to do so said how very much he regretted that Viscount Portman was unable to be present. His lordship had sent them a very kind message, and enclosed them a gift of £500 for which they owed him their most grateful thanks. The speaker mentioned how in 1847 the Samaritan Hospital had consisted only of one small room in Orchid Street! The new out-patient department, which was urgently needed, would have been erected some time ago; but from the fact that the lease of their old building being unexpired, they were unable to move in the matter. Lord Leigh also made very sympathetic reference to the approaching resignation of the matron, Miss C. L. Butler.

The out-patient waiting-room in which the opening ceremony took place was charmingly decorated with a profusion of beautiful flowers, and during the afternoon baskets of these were sold by ladies and children for the benefit of the hospital.

The new building altogether comprises, besides 16 new bedrooms for the nurses, waiting, examination, operation, and surgeons' consulting rooms, and all, of course, replete with modern medical improvements. An entirely new operation table has also been provided. The building, including the furniture for the nurses' bedrooms not yet purchased—£140—will altogether cost £6,140; of this £2,140 has still to be raised.

We should say that the very dainty array of cakes for the visitors' tea on Thursday were all kindly provided by The Oriental Cafés, Limited.

Amongst others present were the Rev. A. W. Oxford, M.A., M.D. (who proposed the vote of thanks unanimously recorded for the presidency of Lord Leigh), Lady Wolseley, Hon. Mrs. Napier, Dr. C. H. Routh, the Hon. Miss Leigh, and a great many of the hospital's nurses.

